# Supplementary material for: Specificity of Escherichia coli Heat-Labile Enterotoxin Investigated by Single-Site Mutagenesis and Crystallography
Source: Int J Mol Sci. 2019 Feb 6;20(3):703. doi: 10.3390/ijms20030703 (PMC6386978; doi:10.3390/ijms20030703)
Supplement: Supplementary file 1 [file ijms-20-00703-s001.pdf]

SUPPLEMENTARY MATERIALS

Specificity of *Escherichia coli* heat-labile enterotoxin investigated by single-site mutagenesis and crystallography

Julie Elisabeth Heggelund [1,#a], Joel Benjamin Heim [1], Gregor Bajc [2], Vesna Hodnik [2,3,#b], Gregor Anderluh [2,3] and Ute Krengel [1,*]

[1] Department of Chemistry, University of Oslo, Postbox 1033 Blindern, 0315 Oslo, Norway; j.e.heggelund@farmasi.uio.no (J.E.H.); j.b.heim@kjemi.uio.no (J.B.H.); ute.krengel@kjemi.uio.no (U.K.)
[2] Department of Biology, Biotechnical Faculty, University of Ljubljana, Jamnikarjeva 101, 1000 Ljubljana, Slovenia; gregor.bajc@bf.uni-lj.si (G.B.); vesna.hodnik@novartis.com (V.H.)
[3] Department of Molecular Biology and Nanobiotechnology, National Institute of Chemistry, Hajdrihova 19, 1000 Ljubljana; Slovenia; gregor.anderluh@ki.si (G.A.)
* Correspondence: ute.krengel@kjemi.uio.no; Tel.: +47-22855461
† Current address: Department of Pharmacy, University of Oslo, Postbox 1068 Blindern, 0316 Oslo, Norway
‡ Current address: Lek d.d., Kolodvorska 27, 1234 Mengeš, Slovenia

Supplementary Materials and Methods

Analysis by Circular Dichroism

Prior to CD analysis, the protein was dialyzed into a 10 mM potassium phosphate buffer at pH 7.4, at a protein concentration of 0.10-0.16 mg/ml. The measurements were carried out using a spectropolarimeter (Jasco J-810) at 2 °C using a quartz cuvette (path length 0.1 cm).

Supplementary Figure

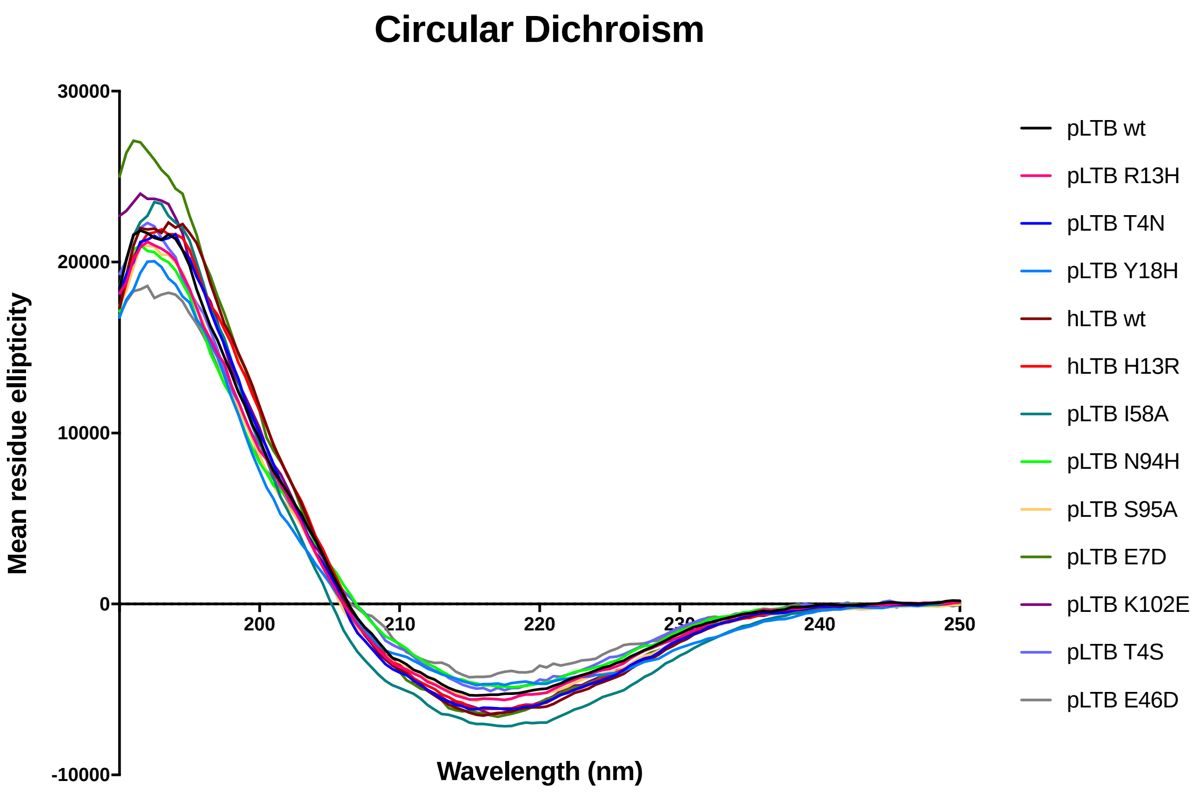

Figure S1. Circular dichroism of pLTB and hLTB variants discussed in the paper, showing that they are folded.
